# Supply Chain Vulnerabilities in First-Line Treatments for Sexually Transmitted Infections: Implications for U.S. Public Health Preparedness

**DOI:** 10.64898/2026.05.06.26352546

**Published:** 2026-05-07

**Authors:** Garcia Coby Y, Leung William, Shirley Amelia, Zhao Ian, Allan-Blitz Lao-Tzu

**Affiliations:** 1 Harvard University, Cambridge, MA, USA; 2 Boston College, Boston, MA, USA; 3 Brigham and Women’s Hospital, Boston, MA, USA; 4 Division of Infectious Diseases, Department of Medicine, University of California, Los Angeles, Los Angeles, CA, USA

**Keywords:** Drug shortages, sexually transmitted infections, antimicrobials, supply chain, public health preparedness

## Abstract

**Objectives:**

To evaluate supply-chain vulnerabilities affecting medications essential for treating sexually transmitted infection in the United States and identify disruption mechanisms that may predispose these therapies to shortages.

**Methods:**

We conducted a qualitative, structured supply-chain vulnerability assessment of first-line medications for five priority sexually transmitted pathogens recommended by the Centers for Disease Control and Prevention and the World Health Organization: azithromycin, doxycycline, ceftriaxone, benzathine penicillin G, metronidazole, tinidazole, acyclovir, and cefixime. Using a predefined framework derived from pharmaceutical supply-chain disruption literature, we evaluated 13 disruption categories spanning raw material sourcing, active pharmaceutical ingredient production, manufacturing, distribution, market dynamics, information systems, and post-distribution loss mechanisms. Each category was assessed using four binary indicators and classified as relevant when at least two criteria were satisfied.

**Results:**

Multiple disruption domains applied across the drug set. Recurrent vulnerabilities included geographically concentrated active pharmaceutical ingredient production, limited manufacturing redundancy in low-margin generic markets, manufacturing constraints affecting sterile injectable products, reliance on consolidated distribution networks, and susceptibility to demand surges and information-system disruptions. All eight drugs exhibited at least one regulatory or market signal consistent with potential supply vulnerability, including documented shortages, product discontinuations, or limited manufacturer participation.

**Conclusions:**

Supply-chain vulnerabilities were identified across multiple first-line sexually transmitted infection therapies, indicating that disruption risk is not confined to a single drug. There is a need for policy interventions to strengthen supply-chain resilience, including diversification of active pharmaceutical ingredient sourcing and distribution networks, as well as incentives for sustainable generic production.

## INTRODUCTION

Access to effective antimicrobial therapies is essential for treating sexually transmitted infections (STIs). The consequences of untreated STIs include pelvic inflammatory disease, infertility, obstetric complications, neonatal death, adverse birth outcomes, and an increased risk for the transmission of HIV ^6^. In June 2023, Pfizer warned that it would soon run out of benzathine penicillin G ^7^. Because Pfizer was the only manufacturer supplying the medication to the United States (U.S.), and because penicillin is the only recommended treatment for syphilis during pregnancy, the shortage created a critical challenge for preventing congenital syphilis ^1,8^. The loss of effective treatment coincided with a resurgence of syphilis and congenital syphilis in the U.S.; since 2012, the number of congenital syphilis cases has increased by more than 1,000% ^9^. The shortage of benzathine penicillin G drew attention to structural weaknesses in the pharmaceutical supply chain with direct consequences for patient care and public health.

Drug shortages arise from vulnerabilities across pharmaceutical supply chains, including upstream sourcing, manufacturing concentration, distribution, and market dynamics ^2,3^. Although prior studies have examined drug shortages broadly, few have focused on STI treatments, many of which are generic, low-margin products with constrained manufacturing bases ^5,10,11^. Whether those drugs share vulnerabilities similar to benzathine penicillin G remains unclear.

In this study, we evaluated supply-chain vulnerabilities across eight core STI drugs recommended by the U.S. Centers for Disease Control and Prevention and the World Health Organization (WHO). Using a structured qualitative framework based on 13 pharmaceutical supply-chain disruption categories, we assessed risks spanning raw materials, manufacturing, distribution, information systems, and market behavior. By grounding our analysis in regulatory data, documented shortage histories, and established disruption mechanisms, we sought to identify which drugs used to treat priority sexually transmitted pathogens may be vulnerable to disruptions analogous to the recent U.S. penicillin shortage and to inform strategies for strengthening supply-chain resilience.

## METHODS

### Research Design

We conducted a qualitative, structured supply-chain vulnerability assessment of core medications used to treat STIs in the U.S. We applied a rule-based framework described below to evaluate whether established pharmaceutical supply-chain disruption factors met predefined criteria for relevance to each drug. We designed the study as a structured qualitative assessment designed to identify recurring categories of supply-chain vulnerability across drugs.

### Drug Selection

We focused on the five priorities STIs identified in the U.S. Sexually Transmitted Infections National Strategic Plan (2021–2025) and its addendum: *Chlamydia trachomatis, Neisseria gonorrhoeae, Treponema pallidum*, *Trichomonas vaginalis*, and herpes simplex virus types 1 and 2. ^12,13^.

We selected the first-line treatment options for each STI recommended by the CDC or WHO STI treatment guidelines as well as acceptable alternative first-line therapies. We excluded therapies recommended only under restricted clinical conditions (e.g., allergy-specific substitutions, salvage regimens for resistant infections, or context-dependent indications).

### Data Collection

We used a structured, multi-source data collection strategy in which each disruption category was evaluated using sources from multiple types of datasets. For peer-reviewed evidence, we searched MEDLINE and Embase for each included drug using predefined combinations of drug names and disruption-related terms. Those searches were designed to identify evidence on formulation characteristics, historical shortages, manufacturing disruptions, distribution constraints, demand surges, recalls, supplier concentration, and related supply-chain vulnerabilities. Full search terms for each drug and concept combination are provided in the Appendix.

For U.S. regulatory and product-market evidence, we reviewed several FDA databases to systematically characterize the structure and resilience of drug supply chains: 1) Drugs@FDA to identify approved new drug applications and abbreviated new drug applications^15^, 2) the FDA Drug Shortages Database to identify active and resolved shortages and manufacturer-reported causes^16^, 3) the Orange Book to assess therapeutic equivalence and approval context^17^, and 4) the National Drug Code Directory to identify listed products and marketed presentations^18^. Together, those sources enabled us to assess key dimensions of supply vulnerability, including manufacturer concentration, approval pathways, shortage history, and marketed-product availability.

For disruption categories that were not systematically indexed or consistently retrievable in bibliographic databases, such as misinformation-driven demand surges, off-label promotion, cyber incidents, trade disputes, and certain distribution shocks, we used a predefined gray-literature search strategy. Specifically, we searched Google search engine as well as specific U.S. government websites: FDA, CDC, Department of Health and Human Services, Administration for Strategic Preparedness and Response, Drug Enforcement Administration, and U.S. Customs and Border Protection when relevant. We also searched international and multilateral websites: WHO and European Medicines Agency.

We included industry and supply-chain sources such as manufacturer-issued recall notices, FDA enforcement reports, drug shortage communications, and publications from major pharmaceutical wholesalers (e.g., AmerisourceBergen, McKesson, and Cardinal Health) and pharmacy organizations (e.g., American Society of Health-System Pharmacists). Finally, we searched the following archival news databases and major news outlets when a disruption event was reported publicly but not yet represented in peer-reviewed or regulatory literature: *The New York Times*, *The Wall Street Journal*, *Reuters*, *Associated Press*, *STAT News*, and *Bloomberg*, as well as LexisNexis and Factiva. We specified the exact website domains, date ranges, and keyword strings used for those searches in the Appendix.

For all sources, we aligned search terms with the corresponding binary assessment questions. For example, searches paired each drug name with terms such as “drug shortage,” “supply chain,” “manufacturing disruption,” “recall,” “API,” “active pharmaceutical ingredient,” “demand surge,” “counterfeit,” “diversion,” “cyberattack,” “export restriction,” and “trade dispute,” as appropriate to the disruption category. We prioritized sources that documented population-level, system-level, or market-level relevance rather than isolated anecdotal events. The Appendix contains the complete search syntax, database interfaces, websites searched, and the decision rules used to determine whether retrieved evidence satisfied a given binary criterion.

### Measures

We evaluated supply-chain vulnerability using disruption categories adapted from prior work, spanning raw material sourcing, manufacturing, distribution, market dynamics, and information systems^14^. For each disruption category, we developed four binary assessment questions designed a priori to determine whether the disruption mechanism was relevant to a given drug. A disruption category was classified as relevant when at least two questions were answered “yes” with documented supporting evidence. Two authors independently evaluated each question. Disagreements were resolved by a third author. The binary classification was used to standardize application of the framework across drugs rather than to quantify the magnitude or severity of vulnerability.

### Ethical Considerations

This study included only publicly available, de-identified databases. As such, it was exempt from institutional review.

## RESULTS

### Identification and Selection of STI Drugs

We identified 14 pharmacologic agents recommended as either first-line or alternative first-line regimens for the five priority STIs (Table 1). Of the 14 identified agents, six were excluded prior to analysis due to therapeutic redundancy (*n* = 2), niche or allergy-restricted use (*n* = 1), salvage therapy for rare resistant infections (*n* = 1), or context-specific alternatives with inconsistent representation across federal databases (*n* = 2) (Table S1). The remaining eight drugs (azithromycin, doxycycline, ceftriaxone, cefixime, benzathine penicillin G, metronidazole, tinidazole, and acyclovir) comprised the final analytic sample used for structured supply-chain vulnerability assessment (Table 2).

### Regulatory Status, Manufacturer Landscape, and Shortage History

Table 2 summarizes regulatory approval status, formulation type, commercially active U.S. manufacturers, and documented shortage or discontinuation history for the eight included drugs. Six drugs were oral solid-dose generics (azithromycin, doxycycline, cefixime, metronidazole, tinidazole, and acyclovir). Two drugs, ceftriaxone and benzathine penicillin G, were sterile injectable formulations requiring aseptic manufacturing and parenteral administration.

Manufacturer participation varied substantially across drugs. Oral generics were produced by multiple manufacturers, whereas benzathine penicillin G and the oral suspension packets of Azithromycin had a single primary U.S. supplier, indicating high manufacturing concentration. Ceftriaxone had six identified manufacturers, and the other six drugs had more than one U.S. supplier.

FDA Drug Shortages Database records documented prior shortages for benzathine penicillin G (shortage posted April 2023) and metronidazole (shortage posted January 2022), with additional recall and supply disruption events noted for benzathine penicillin G in 2025 (Table 3). No active or historical shortage entries were identified for cefixime, tinidazole, or acyclovir. Ceftriaxone had no active class-wide shortage listing at the time of review despite its reliance on sterile manufacturing.

### Supply-Chain Vulnerability Framework

Application of the supply-chain disruption framework identified vulnerabilities across multiple stages of the pharmaceutical supply chain, including active pharmaceutical ingredient sourcing, sterile manufacturing processes, distribution systems, market structure, and demand variability (Table 3).

Sterile injectable drugs, ceftriaxone and benzathine penicillin G, require aseptic manufacturing, sterile fill–finish production, and specialized packaging components such as sterile vials or prefilled syringes. Those manufacturing requirements necessitate additional production stages and quality-control processes not present in oral solid-dose manufacturing. Oral antibiotics including azithromycin, doxycycline, metronidazole, and cefixime relied on globally distributed ingredient manufacturing and multi-stage supplier networks.

All eight drugs relied on globally distributed active pharmaceutical ingredient supply chains, with ≥50% of active pharmaceutical ingredient volume manufactured outside the U.S. or European Union (Table 3). Upstream sourcing networks involving multiple intermediaries (≥3) were identified for all drugs, indicating widespread exposure to multi-step supply dependencies.

Market structure vulnerabilities were observed across the generic drug set. All eight drugs were generic products distributed through major U.S. pharmaceutical wholesalers and characterized by documented evidence of sustained price erosion or manufacturer exit (Table 3). Evidence of pricing irregularities during shortages was identified for azithromycin and doxycycline, while benzathine penicillin G demonstrated extreme supplier concentration (≤3 active pharmaceutical ingredient manufacturers supplying ≥75% of demand).

Demand-side vulnerabilities were also identified. Evidence from regulatory communications, media reports, and prior studies suggested misinformation-related or off-label demand surges was documented for azithromycin, doxycycline, cefixime, and tinidazole, including instances where social media activity promoted unsupported uses and associated with reported increases in utilization, prompting regulatory warnings and, in some cases, contributing to supply disruptions (Table 3).

Across distribution and system-level domains, evidence consistent with vulnerabilities related to transportation disruptions, cyberattacks, and diversion was identified across all drugs, supported by documented incidents such as shipment delays, cyberattacks on pharmaceutical manufacturers and distributors, and diversion within supply chains.

The binary classification matrix summarizing disruption categories for each drug is presented in Table 3. This matrix represents an exploratory application of the framework and does not provide a quantitative ranking of drug-specific vulnerability.

## DISCUSSION

We conducted a structured supply-chain vulnerability assessment of eight first-line therapies used to treat priority STIs in the U.S. Our analysis identified multiple supply-chain vulnerabilities across those drugs, including internationally concentrated active pharmaceutical ingredient production, manufacturing complexity in sterile injectable formulations, economic pressures within generic drug markets, and emerging disruption pathways, including misinformation-driven demand surges and cybersecurity-related disruptions affecting pharmaceutical distribution systems.

The recent U.S. shortage of benzathine penicillin G demonstrated how structural fragilities in generic pharmaceutical supply chains can rapidly translate into public health consequences ^1^. Prior studies of drug shortages consistently identify manufacturing concentration, thin profit margins, and limited production redundancy as central drivers of supply instability, particularly for sterile injectable generics ^3,19,20^. Our findings suggest that those structural characteristics may extend beyond benzathine penicillin G to multiple first-line STI treatments.

Interruptions in the supply of STI treatments can have direct clinical and public health consequences. Untreated chlamydia and gonorrhea can lead to pelvic inflammatory disease, infertility, ectopic pregnancy, and chronic pelvic pain ^6^. Trichomoniasis, syphilis, and genital herpes are associated with adverse pregnancy and neonatal outcomes including preterm birth, miscarriage, stillbirth, neonatal death, and congenital infection ^6^. All of those STIs have been associated with increased HIV transmission risk.^6^ Reduced availability of effective therapies may force use of less effective or more toxic regimens, lowering cure rates and increasing transmission ^21^. Because STI control depends on rapid diagnosis and treatment, sustained drug availability is critical to prevention infrastructure.

Antibiotic shortages are not new. Penicillin G shortages have been documented since the 1970s and have frequently been associated with manufacturing transitions, facility closures, or limited producer redundancy ^22^. Over time, localized disruptions have become embedded within globally distributed pharmaceutical supply networks. Contemporary shortages increasingly reflect geographically concentrated active pharmaceutical ingredient manufacturing and reliance on a limited number of production facilities, creating single-point failure risks ^22,23^. Our analysis identified reliance on internationally distributed active pharmaceutical ingredient manufacturing across several drugs evaluated in this study. More than one quarter of active pharmaceutical ingredient manufacturing facilities supplying the U.S. market are domestic, while 72% are located overseas, with substantial shares in China (13%) and India (18%) ^24–27^. Outsourcing of active pharmaceutical ingredient production for antibiotics to Asia has intensified that geographic concentration over recent decades ^28,29^.

Trade patterns further highlight the uneven structure of pharmaceutical supply chains. Whereas benzathine penicillin G depends on a highly specialized and fragile production network, often limited to a small number of upstream suppliers and prone to recurring global shortages, azithromycin is produced within a broad, diversified generic market that supports large-scale, reliable distribution. By 2016, just three Chinese active pharmaceutical ingredient manufacturers supplied the global penicillin market, whereas azithromycin was among the most frequently prescribed antibiotics, accounting for 26.3% of local purchases and 73.7% from foreign sources ^30,31^. That contrast illustrates how concentrated production may create persistent supply vulnerabilities, while competitive manufacturing enhances resilience. Adding to this complexity, India remains dependent on Chinese upstream chemical inputs for pharmaceutical ingredients used in the production of many generic antibiotics, including azithromycin; this interdependence introduces additional exposure to bilateral trade disruptions affecting antibiotic supply chains ^32^.

Supply disruptions in concentrated manufacturing systems may propagate rapidly. For example, contamination in a single Italian manufacturing facility combined with raw material shortages in China threatened nearly 60% of Japan’s cefazolin supply ^33^. Limited transparency regarding active pharmaceutical ingredient supplier identity and production capacity further complicates supply-chain monitoring. Manufacturers may rely on intermediaries and therefore lack direct visibility into upstream ingredient sources, reducing the ability of regulators or purchasers to detect emerging supply bottlenecks ^2^. Those structural characteristics specifically affected azithromycin, doxycycline, metronidazole, and cefixime ^34^.

Sterile injectable drugs present additional manufacturing constraints. Ceftriaxone and benzathine penicillin G require aseptic manufacturing, sterile fill–finish production, and specialized packaging components. Those processes are technically complex, capital intensive, and sensitive to quality failures, factors that frequently contribute to drug shortages ^3^. Ceftriaxone injection products have experienced recalls due to particulate contamination, including rubber particles originating from vial stoppers, illustrating how failures in packaging or aseptic processing can interrupt supply even when active pharmaceutical ingredient availability remains stable ^35^. National analyses indicated that parenteral drugs accounted for 71.7% of shortages in critical care medications over a 15-year period ^36^.

Economic characteristics of generic pharmaceutical markets also contribute to supply instability. Many STI treatments evaluated in this study are long-established generics with limited pricing flexibility and narrow profit margins. An economic analysis found that low profit margins, price volatility, and manufacturer exit from unprofitable therapeutic classes reduced production redundancy and increased the likelihood of shortages ^37^. Procurement systems that emphasize lowest-cost purchasing may further reduce incentives for sustained manufacturing capacity ^38^.

Emerging disruption mechanisms also affect pharmaceutical supply systems. Demand surges driven by misinformation or off-label prescribing can rapidly destabilize supply ^39^. During the COVID-19 pandemic, increased off-label use of azithromycin produced documented supply distortions in several markets ^40^. For example, the azithromycin market in Brazil experienced a profound supply distortion, with consumption surging by 62.8% due to political endorsements and off-label prescribing “COVID kits” ^41^. Cybersecurity incidents pose additional risks; attacks on healthcare logistics systems have disrupted medication ordering and distribution networks in multiple settings ^42^. The WHO estimated that approximately 10% of medical products in low- and middle-income countries are substandard or falsified, with antibiotics among the most affected categories ^43^. Digital traceability systems, including blockchain-based distribution networks, have been proposed to improve supply transparency and reduce counterfeit circulation ^44^. Although regulatory oversight is stronger in the U.S., online purchasing channels and international supply networks may still introduce counterfeit or diverted medicines into the market ^45^.

Taken together, our findings indicate that vulnerabilities affecting benzathine penicillin G are not unique to a single drug but arise from broader structural characteristics of generic pharmaceutical supply chains. Supply instability reflects interactions between manufacturing concentration, global ingredient sourcing, market incentives, regulatory oversight, and distribution infrastructure. Addressing those vulnerabilities may require coordinated policy interventions, including diversification of active pharmaceutical ingredient sourcing, increased manufacturing redundancy, improved supply-chain transparency, and economic incentives that support sustainable generic production capacity ^46^. Stronger supply chain integration and coordination are associated with improved resilience under disruption conditions ^10^. Strengthening supply-chain resilience is therefore an important component of maintaining reliable access to essential STI treatments.

From a public health practice perspective, those findings suggest several actionable strategies. First, health systems and public health agencies should monitor supplier concentration for essential antimicrobials and prioritize procurement from diversified manufacturers. Second, policymakers should consider incentives for domestic or geographically distributed active pharmaceutical ingredient production to reduce reliance on concentrated global sources. Third, surveillance systems could incorporate early warning indicators such as demand surges and manufacturer exits. Finally, integrating supply-chain risk assessments into STI control programs may improve preparedness for future disruptions.

### Limitations

This study has several limitations. First, the framework used a binary classification approach rather than quantitative risk scoring. While that permitted consistent comparison across drugs, it precluded estimating the magnitude or probability of disruption. Second, some disruption categories, such as misinformation-driven demand surges and cybersecurity incidents, were not systematically captured in regulatory or bibliographic databases. Evidence for those mechanisms relied partly on gray literature and event reporting, which may be incomplete or uneven across drugs and could lead to under-identification of certain vulnerabilities. Third, our analysis relied on qualitative synthesis of regulatory records, published literature, and documented supply-chain events, which limits causal inference between identified vulnerabilities and observed or future drug shortages. Fourth, the binary classification approach may oversimplify heterogeneity in the severity and likelihood of disruption across drugs and categories. Future research incorporating quantitative market concentration metrics, geographic mapping of active pharmaceutical ingredient production, and longitudinal shortage data could improve risk estimation and modeling of supply-chain disruption.

## Conclusion

Multiple structural vulnerabilities affect eight first-line therapeutics for the five priority sexually transmitted pathogens, including reliance on internationally distributed active pharmaceutical ingredient production, manufacturing constraints associated with sterile injectable formulations, market concentration within generic drug manufacturing, and documented disruption events affecting pharmaceutical supply systems.

## Supplementary Material

Supplement 1

## Figures and Tables

**Table 1.** First-Line and Alternative First-Line Pharmacologic Treatments for Priority Sexually Transmitted Infections, United States, 2021–2025

Pathogen	First-line treatment (drug, dose, duration)	Second-line / Alternate treatment(s)	Notes / Special considerations
*Chlamydia trachomatis* ^47–51^	Doxycycline 100 mg orally twice daily for 7 days	Azithromycin 1 g orally as a single dose; Levofloxacin 500 mg orally once daily for 7 days	In pregnancy: azithromycin or amoxicillin recommended. Doxycycline should be avoided during pregnancy. Retesting recommended after approximately 3 months due to reinfection risk.
*Neisseria gonorrhoeae* ^47,52,53^	Ceftriaxone 500 mg intramuscular injection as a single dose for persons weighing less than 150 kilograms; 1 g for persons weighing 150 kilograms or more	Cephalosporin allergy: gentamicin 240 mg intramuscular injection plus azithromycin 2 g orally as a single dose; if ceftriaxone unavailable: cefixime 800 mg orally as a single dose	Evaluate and treat possible chlamydia coinfection if not excluded. In pregnancy, ceftriaxone is recommended.
*Treponema pallidum* ^47,50,54^	Benzathine penicillin G 2.4 million units intramuscular injection as a single dose for primary, secondary, or early latent infection	Late latent infection or unknown duration: benzathine penicillin G 2.4 million units intramuscular injection weekly for three doses. Neurosyphilis: aqueous crystalline penicillin G intravenous therapy for 10–14 days	Penicillin is the only recommended therapy for syphilis during pregnancy and congenital infection. Penicillin allergy usually requires desensitization.
*Trichomonas vaginalis* ^47,55^	Women: Metronidazole 500 mg orally twice daily for 7 days; Men: Metronidazole 2 g orally as a single dose	Tinidazole 2 g orally as a single dose; higher-dose regimens for resistant infection	Sexual partners should receive treatment concurrently to prevent reinfection.
*Herpes Simplex Virus* ^47,56^	First clinical episode: Acyclovir 400 mg orally three times daily for 7–10 days; Famciclovir 250 mg orally three times daily for 7–10 days; Valacyclovir 1 g orally twice daily for 7–10 days	Suppressive therapy: Acyclovir, valacyclovir, or famciclovir daily regimens; Episodic therapy for recurrent outbreaks	Suppressive therapy reduces transmission risk. Management includes counseling and avoiding sexual contact during active lesions.

**Table 2.** Regulatory Status, Formulation Type, Manufacturer Participation, and Shortage History of Eight STI Medications, United States, 2022–2025

Drug (Primary STI indication)	Formulation focus	Regulatory labelers	Commercial U.S. suppliers	FDA shortage status^57^	Latest shortage or recall note	CDC substitute guidance
**Acyclovir** (genital herpes)	Oral tablets and capsules	Multiple	Multiple	Discontinuation noted (topical formulation)	Topical acyclovir discontinuation reported March 2025; oral formulations remain available	Acyclovir, valacyclovir, and famciclovir are recommended options for episodic or suppressive therapy
**Azithromycin** (alternative for chlamydia; pregnancy use)	Option 1: Oral tablet Option 2: 1 g oral suspension packets	Option 1: Multiple Option 2: Pfizer (1 g packets)	Option 1: Multiple Option 2: 1, Pfizer	Injection discontinued	Intravenous azithromycin 500 mg discontinued September 2025; oral formulations remain available	Alternative regimen for chlamydia when doxycycline cannot be used
**Benzathine penicillin G** (syphilis treatment in adults and pregnancy; not used for congenital infection)	Intramuscular depot suspension (prefilled syringes)	Pfizer / King Pharmaceuticals (Bicillin L-A)	1	Shortage (posted April 26, 2023)	Recall of selected Bicillin L-A lots reported July 2025; temporary importation routes implemented during shortage	No substitute for syphilis during pregnancy; penicillin required (desensitize if allergic)
**Cefixime** (oral alternative for gonorrhea when ceftriaxone cannot be administered)	Oral tablets and capsules	Multiple	Multiple	No active shortage listing	No current FDA shortage or recall entry identified	Cefixime 800 mg orally once if ceftriaxone cannot be administered; ensure chlamydia coverage
**Ceftriaxone** (first-line treatment for gonorrhea; also used in pelvic inflammatory disease regimens)	Sterile parenteral (lyophilized powder or premix)	Apotex; Fresenius Kabi; Hikma; Pfizer; Piramal Critical Care; Agent	6	No active shortage listing	No current classwide shortage entry in the FDA Drug Shortages database	Ceftriaxone remains the recommended first-line therapy
**Doxycycline** (first-line treatment for chlamydia)	Oral tablets and capsules	Multiple	Multiple	Discontinuations reported	No active shortage listing identified in FDA Drug Shortages database	First-line regimen: doxycycline 100 mg orally twice daily for 7 days; azithromycin alternative
**Metronidazole** (trichomoniasis; also used in pelvic inflammatory disease regimens)	Oral tablets; intravenous bags	Multiple	Multiple	Shortage (posted January 13, 2022)	Causes include active ingredient shortage and manufacturing discontinuations	Standard regimen: metronidazole 500 mg orally twice daily for 7 days; tinidazole alternative
**Tinidazole** (alternative for trichomoniasis)	Oral tablets	Multiple	Multiple	No active shortage listing	No current FDA shortage or recall entry identified	Alternative regimen for trichomoniasis

**Table 3.** Binary Classification of Supply-Chain Vulnerabilities Across Eight First-Line STI Medications Using a 13-Category Disruption Framework, United States, 2022–2025

Category	Question	Drug							
		Acyclovir ^32,58–64^	Azithromycin ^4,5,26,65–78^	Benzathine penicillin G ^1,11,29,79–82^	Ceftriaxone ^5,35,67,79,83–91^	Cefixime ^83,92–99^	Doxycycline ^4,95,100–109^	Metronidazole ^4,32,67,79,95,110–117^	Tinidazole ^32,67,118–125^
1. Raw Material Adulteration	Do any critical raw materials used in the US-market version of this drug in its more common form originate from animal, microbial, or agricultural sources?	N	Y	Y	Y	Y	Y	N	N
	In the past 15 years, have critical raw materials used in this drug or drug class been sourced from regions with documented FDA, European Medicines Agency, or WHO regulatory enforcement limitations?	Y	Y	Y	Y	Y	Y	Y	Y
	Is upstream sourcing for critical raw materials fragmented across ≥3 intermediaries before reaching U.S.-based API or finished-dose manufacturers?	Y	Y	Y	Y	Y	Y	Y	Y
	In the past 15 years, have FDA recalls, import alerts, or safety communications cited raw-material contamination for this drug or drug class in the U.S.?	N	N	N	N	N	N	N	N
2. API Adulteration	Is the API for the U.S. market produced by ≤3 manufacturers that collectively supply ≥75% of U.S. demand?	N	N	Y	N	N	N	N	N
	Is ≥50% of the API volume for the U.S. market manufactured outside the U.S. or EU?	Y	Y	Y	Y	Y	Y	Y	Y
	Has the API experienced impurity-related recalls?	N	N	N	N	N	N	N	N
	Does the documented API synthesis pathway involve reaction steps known to generate toxic or genotoxic by-products (per FDA/ International Council for Harmonization of Technical Requirements for Pharmaceuticals for Human Use guidance)?	Y	Y	N	N	N	Y	N	N
3. Excipient Adulteration	Does the most common U.S.-market STI formulation, in the form immediately preceding consumption or administration, rely on excipients with a documented history of adulteration (e.g., Diethylene Glycol, melamine) in FDA or U.S. Pharmacopeia records?	N	N	N	N	N	Y	N	N
	Do excipients constitute >50% of the finished product mass or volume for the STI-indicated formulation?	Y	Y	Y	Y	Y	N	N	Y
	Are excipients regulated under a weaker oversight framework than APIs (e.g., no Drug Master File requirement) for this product?	N	N	N	N	N	Y	N	N
	In the past 15 years, have FDA alerts or recalls implicated excipients used in this drug or drug class?	N	N	N	N	N	N	N	N
4. Counterfeiting	Is the STI-indicated formulation commonly distributed internationally or sold via online pharmacies accessible to U.S. consumers?	Y	Y	Y	Y	Y	Y	Y	Y
	Has the dosage form (e.g., tablet, vial, suspension) been identified in regulatory or enforcement reports as susceptible to replication or relabeling?	N	N	N	N	N	Y	N	N
	In the past 15 years, has the drug (STI indication) been identified by FDA, U.S. Department of Justice, or U.S. law-enforcement agencies as counterfeit-prone?	N	N	Y	Y	N	Y	Y	N
	Have regulatory reports (e.g., FDA or WHO) documented counterfeit or substandard versions of this drug that required laboratory testing for confirmation rather than visual or packaging inspection alone?	Y	Y	Y	Y	Y	Y	Y	Y
5. Internal Losses	Is the drug or API stored in bulk or high-value quantities at U.S. manufacturing or distribution facilities?	N	N	Y	Y	N	Y	Y	N
	Are materials stored across multiple internal locations within a facility (e.g., multiple warehouses or subcontractors)?	N	N	Y	Y	N	Y	Y	N
	In the past 15 years, has internal theft or unexplained loss been documented for this drug or drug class?	N	N	N	N	N	N	N	N
	Have internal losses or theft been associated with documented supply disruptions for this drug or drug class in the U.S.?	N	N	N	N	N	N	N	N
6. External Losses	Does U.S. manufacturing of this drug generate recoverable waste materials (e.g., solvents, intermediates, packaging)?	Y	N	Y	Y	Y	Y	Y	Y
	Are disposal or waste-handling processes for the U.S. market outsourced to third-party contractors?	N	N	N	N	N	N	N	N
	In the past 15 years, has diversion from pharmaceutical waste streams been documented for this drug or drug class?	N	N	N	N	N	Y	N	N
	Have regulatory reports or published studies documented diversion, reuse, or reintroduction of discarded materials from this drug or drug class into licit or illicit supply chains?	Y	N	Y	Y	Y	N	Y	Y
7. Transportation Losses	Is the most common STI-indicated formulation for the U.S. shipped in bulk or high-value quantities?	Y	Y	Y	Y	Y	Y	Y	Y
	Does the most common STI-indicated formulation require cold-chain or specialized transport for U.S. distribution?	N	N	Y	Y	N	N	N	N
	In the past 15 years, have transportation thefts or losses been reported for this drug or drug class affecting the U.S. market?	Y	Y	Y	Y	Y	Y	Y	Y
	Have transportation disruptions or losses been associated with documented delays, shortages, or substitutions for this drug or drug class in the U.S.?	Y	Y	Y	Y	Y	Y	Y	Y
8. Diversion	Is the drug used in U.S. public, subsidized, or discounted STI treatment programs?	Y	N	Y	Y	Y	Y	Y	Y
	Does the drug have documented resale value in U.S. secondary or informal markets?	N	N	N	N	N	N	N	N
	In the past 15 years, has diversion been documented for this drug or drug class in the U.S.?	Y	Y	Y	Y	Y	Y	Y	Y
	Is end-to-end tracking from wholesaler to patient absent or not routinely implemented for this STI formulation?	Y	Y	Y	Y	Y	Y	Y	Y
9. Cyberattacks	Does production or distribution for the U.S. market rely on digitally integrated manufacturing systems?	Y	Y	Y	Y	Y	Y	Y	Y
	Are key API or finished-dose suppliers integrated into shared IT or data platforms?	Y	Y	Y	Y	Y	Y	Y	Y
	In the past 15 years, have cyberattacks affected manufacturers or distributors in this drug class?	Y	Y	Y	Y	Y	Y	Y	Y
	Have cyberattacks or system outages affecting manufacturers or distributors resulted in documented disruptions to production or distribution for this drug or drug class?	Y	Y	Y	Y	Y	Y	Y	Y
10. Wholesaler Price Discrimination	Is the drug primarily distributed through major U.S. wholesalers (e.g., Big Three)?	Y	Y	Y	Y	Y	Y	Y	Y
	Is the drug a generic with documented evidence of sustained price erosion or manufacturer exit in the U.S. market?	Y	Y	Y	Y	Y	Y	Y	Y
	In the past 15 years, have pricing irregularities been documented during U.S. shortages?	N	Y	N	N	N	Y	N	N
	Have pricing practices (e.g., price spikes, allocation restrictions) been associated with documented access limitations or supply disruptions for this drug during U.S. shortages?	Y	Y	Y	Y	Y	Y	Y	Y
11. Disinformati on Campaigns	In the past 15 years, has the drug been promoted in the U.S. for unsupported or off-label uses?	Y	Y	N	N	Y	Y	N	Y
	Is there documented evidence that U.S. social media activity has measurably influenced demand for this drug?	Y	Y	N	N	Y	Y	N	Y
	Have FDA, CDC, or Federal Trade Commission issued misinformation warnings related to this drug?	Y	Y	N	N	N	Y	N	Y
	Have demand surges (e.g., from off-label use or misinformation) been associated with documented supply disruptions or shortages for this drug or drug class?	Y	Y	Y	Y	Y	Y	Y	Y
12. Illicit Reverse Logistics	Does the drug, in its most common form, retain potency after expiration under typical storage conditions?	Y	Y	Y	Y	N	Y	Y	Y
	Is the drug frequently returned, unused, or discarded in U.S. healthcare settings?	Y	Y	Y	Y	Y	Y	Y	Y
	In the past 15 years, has resale of expired drugs been documented for this drug or drug class?	Y	Y	Y	Y	Y	Y	Y	Y
	Are reverse-logistics pathways for this drug subject to limited regulatory oversight (e.g., absence of standardized tracking or reporting requirements)?	Y	Y	Y	Y	Y	Y	Y	Y
13. Intellectual Property Theft	Is the drug still under patent or regulatory exclusivity in the U.S.?	N	N	N	N	N	N	N	N
	Does manufacturing rely on proprietary methods or formulations?	N	N	N	N	N	Y	N	N
	In the past 15 years, has IP theft occurred in this therapeutic area (i.e. antibiotics for STI treatment)?	Y	Y	Y	Y	Y	Y	Y	Y
	Have regulatory reports or studies identified this drug or drug class as vulnerable to counterfeit production due to accessible formulations or manufacturing knowledge?	N	N	N	N	N	N	N	N

## Data Availability

Data supporting this study is available upon request. A detailed spreadsheet containing the data, along with source links, can be accessed by contacting the corresponding author.

## ABREVATIONS

FDA: U.S. Food and Drug Administration
HIV: Human Immunodeficiency Virus
WHO: World Health Organization
CDC: Centers for Disease Control and Prevention
STI: Sexually Transmitted Infection
API: Active Pharmaceutical Ingredient
U.S.: United States
EU: European Union
