# Supplementary material for: Supply Chain Vulnerabilities in First-Line Treatments for Sexually Transmitted Infections: Implications for U.S. Public Health Preparedness
Source: medRxiv. 2026 May 7:2026.05.06.26352546. Preprint. [Version 1] doi: 10.64898/2026.05.06.26352546 (PMC13174745; doi:10.64898/2026.05.06.26352546)
Supplement: Supplement 1 [file NIHPP2026.05.06.26352546v1-supplement-1.pdf]

medRxiv preprint doi: https://doi.org/10.64898/2026.05.06.26352546; this version posted May 7, 2026. The copyright holder for this preprint (which was not certified by peer review) is the author/funder, who has granted medRxiv a license to display the preprint in perpetuity.
It is made available under a CC-BY 4.0 International license .

APPENDIX

Drug to Exclude	Primary Reason for Exclusion	Detailed Context
Valacyclovir	Prodrug/Redundancy	Delivers the same active ingredient as Acyclovir. Exclusion simplifies the list by removing therapeutic duplication.
Gentamicin	Niche Alternate/Allergy	Only used for Gonorrhea as a second-line option for patients with severe cephalosporin allergy.
Foscarnet	Salvage Therapy/Rare Use	Reserved for rare, drug-resistant Herpes cases, typically in immunocompromised patients; far outside standard treatment.
Levofloxacin	Non-Standard Alternate	A Fluoroquinolone antibiotic used as a less-preferred alternative for Chlamydia.
Amoxicillin	Niche Use/Pregnancy	Used only as a specific, second-choice alternative for Chlamydia treatment in pregnancy.
Famciclovir	Prodrug/Redundancy	Like Valacyclovir, it's an alternative antiviral prodrug for Herpes, which is covered by Acyclovir.

Table S1. Characteristics of Excluded Pharmacologic Agents and Reasons for Exclusion from Supply-Chain Vulnerability Analysis, United States, 2021–2025

medRxiv preprint doi: https://doi.org/10.64898/2026.05.06.26352546; this version posted May 7, 2026. The copyright holder for this preprint (which was not certified by peer review) is the author/funder, who has granted medRxiv a license to display the preprint in perpetuity. It is made available under a CC-BY 4.0 International license .

Search Strategy, Evidence Sources, and Binary Assessment Framework

1. Bibliographic Database Search Strategy

Peer-reviewed evidence was identified through structured searches of MEDLINE (via PubMed) and Embase (via Elsevier Embase interface). Searches were conducted between January 1, 2015 and January 31, 2026. For each drug included in the analytic sample (acyclovir, azithromycin, benzathine penicillin G, cefixime, ceftriaxone, doxycycline, metronidazole, and tinidazole), searches combined the generic drug name with disruption-related terms designed to identify evidence of pharmaceutical supply-chain vulnerabilities.

The core search syntax was: ("drug name") AND ("drug shortage" OR "supply chain" OR "manufacturing disruption" OR "manufacturing quality issue" OR "recall" OR "active pharmaceutical ingredient" OR "API shortage" OR "production capacity" OR "supply disruption" OR "distribution disruption" OR "demand surge" OR "counterfeit" OR "diversion" OR "cyberattack" OR "export restriction" OR "trade dispute")

Searches were limited to English-language publications. Search results were screened to identify evidence describing all 13 categories.

2. U.S. Regulatory and Market Databases

To characterize regulatory status and market structure, we reviewed the following databases:

- **a.** Drugs@FDA: Used to identify approved New Drug Applications (NDAs) and Abbreviated New Drug Applications (ANDAs).
- **b.** FDA Drug Shortages Database: Used to identify (1) active shortages, (2) resolved shortages, (3) manufacturer-reported causes of shortage, and (4) estimated resupply timelines.
- **c.** Approved Drug Products with Therapeutic Equivalence Evaluations (Orange Book): Used to assess: (1) therapeutic equivalence codes, (2) approval history, and (3) application holders.
- **d.** National Drug Code Directory: Used to identify: (1) marketed products, (2) dosage forms, (3) packaging presentations and (4) active labelers.

These databases were accessed in December 2025.

medRxiv preprint doi: https://doi.org/10.64898/2026.05.06.26352546; this version posted May 7, 2026. The copyright holder for this preprint (which was not certified by peer review) is the author/funder, who has granted medRxiv a license to display the preprint in perpetuity. It is made available under a CC-BY 4.0 International license .

3. Gray Literature and Event Detection Strategy

Certain disruption categories, such as misinformation-driven demand surges, cyber incidents, and trade disputes, are not traditionally systematically indexed in bibliographic databases. To identify evidence for these domains, we conducted structured gray-literature searches. Searches were performed using the Google search engine and targeted website queries.

a. U.S. Government Sources: The following domains were searched: The following domains were searched: fda.gov, cdc.gov, hhs.gov, aspr.hhs.gov, dea.gov, cbp.gov.
b. International and Multilateral Organizations: The following domains were searched: who.int, oecd.org, worldbank.org.
c. Industry and Supply-Chain Sources: Sources reviewed included manufacturer recall notices, pharmaceutical wholesaler communications, and pharmacy-organization publications when directly relevant to the assessment question.
d. News and Archival Sources: To identify publicly reported disruption events, we searched: Archival and media sources included Reuters, Bloomberg, major national newspapers, and LexisNexis or similar news databases where available. These sources were used to identify publicly reported events such as manufacturing shutdowns, contamination events, export restrictions, trade disputes, supply disruptions, and cybersecurity incidents affecting pharmaceutical distribution systems.

4. Gray Literature Search Syntax

Searches were conducted between January 2024 and January 2026 The core search syntax was: ("drug name") AND ("drug shortage" OR "supply chain" OR "manufacturing disruption" OR "manufacturing quality issue" OR "recall" OR "active pharmaceutical ingredient" OR "API shortage" OR "production capacity" OR "supply disruption" OR "distribution disruption" OR "demand surge" OR "counterfeit" OR "diversion" OR "cyberattack" OR "export restriction" OR "trade dispute")

5. Review Process

Three reviewers independently evaluated each drug–disruption category pair using a standardized evidence extraction log. For each binary question, reviewers documented: (1) source, (2) supporting evidence, and (3) classification (yes or no). Disagreements were resolved through discussion and consensus review.
